# PW03-007 - NLRP3 genetic variants in Schnitzler’s syndrome

**DOI:** 10.1186/1546-0096-11-S1-A233

**Published:** 2013-11-08

**Authors:** HD De Koning, J Schalkwijk, JW van der Meer, PL Zeeuwen, K Neveling, M van Gijn, A Simon

**Affiliations:** 1 Dermatology, Radboud University Nijmegen Medical Centre, Nijmegen, Netherlands; 2 Internal Medicine, Radboud University Nijmegen Medical Centre, Nijmegen, Netherlands; 3 Genetics, Radboud University Nijmegen Medical Centre, Nijmegen, Netherlands; 4 Genetics, UMC Utrecht, Utrecht, Netherlands

## Introduction

Schnitzler’s syndrome (SchS) is an autoinflammatory disorder, characterized by chronic urticaria, fever, gammopathy and bone pain. The etiology is unknown, but interleukin-1 (IL-1) inhibition is very effective, like in the cryopyrin associated periodic syndrome (CAPS), that is caused by activating *NLRP3* mutations. Previously, a V198M mutation in *NLRP3* was reported in one patient with SchS, but this is a prevalent variation in the general healthy population.

## Objectives

To study presence and significance of *NLRP3* genetic variants in SchS.

## Methods

We performed exome screening on peripheral blood-derived DNA of three patients with SchS, and Sanger sequencing of *NLRP3* on peripheral blood-derived DNA of 9 patients with SchS. Patients were further clinically characterized and cytokine stimulation studies with peripheral blood mononuclear cells (PBMCs) were performed.

## Results

We found *NLRP3* genetic variants in two patients. Exome screening revealed the known pathogenic CAPS-causing *NLRP3* c.1575C>G p.(P525L) mutation in one patient. Confirmation by Sanger sequencing on peripheral blood only showed a small aberrant peak at the corresponding location. In another patient, we found a hitherto unknown *NLRP3* variant c.1303A>G p.(K435E), of which the pathogenicity still needs to be determined. None of the patients had clinically affected family members. No V198M mutation in *NLRP3* was detected in our population of SchS.

The two patients with *NLRP3* variants fulfilled the criteria for SchS, and had the most severe clinical phenotype of the group. Also, both patients had IgG instead of IgM gammopathy, and both patients had the highest production of IL-1 and IL-6 upon stimulation of PBMCs with LPS.

## Conclusion

In seven of nine patients with SchS, no *NLRP3* mutations were found. Two patients with IgG-type SchS with a severe phenotype carried a genetic variation in the *NLRP3* gene: in one, the novel variant K435E, and in the other one a known mutation P525L that was described in severe CAPS patients.

We hypothesize that somatic mosaicism or a less pathogenic effect of the novel mutation may explain the late onset of symptoms.

## Disclosure of interest

None declared

